# Rad1 attenuates DNA double-strand breaks and cell cycle arrest in type II alveolar epithelial cells of rats with bronchopulmonary dysplasia

**DOI:** 10.1186/s10020-023-00660-3

**Published:** 2023-05-24

**Authors:** Xin Tong, Danni Li, Na Liu, Wanjie Huang, Xinyi Zhao, Dan Zhang, Xindong Xue, Jianhua Fu

**Affiliations:** Department of Pediatrics, Shengjing Hospital of China Medical University, Shenyang, China

**Keywords:** Bronchopulmonary dysplasia, Arrested lung development, DNA double-strand breaks, Rad1, Cell cycle arrest

## Abstract

**Background:**

Bronchopulmonary dysplasia (BPD) is the most common and serious chronic lung disease in preterm infants with pathological characteristics of arrested lung development. DNA double-strand breaks (DSBs) are a serious manifestation of oxidative stress damage, but little is known about the role of DSBs in BPD. The current study set out to detect DSB accumulation and cell cycle arrest in BPD and study the expression of genes related to DNA damage and repair in BPD through DNA damage signaling pathway-based PCR array to determine a suitable target to improve arrested lung development associated with BPD.

**Methods:**

DSB accumulation and cell cycle arrest were detected in a BPD animal model and primary cells, then a DNA damage signaling pathway-based PCR array was used to identify the target of DSB repair in BPD.

**Results:**

DSB accumulation and cell cycle arrest were shown in BPD animal model, primary type II alveolar epithelial cells (AECII) and cultured cells after exposure to hyperoxia. Of the 84 genes in the DNA damage-signaling pathway PCR array, eight genes were overexpressed and 11 genes were repressed. Rad1, an important protein for DSB repair, was repressed in the model group. Real-time PCR and western blots were used to verify the microarray results. Next, we confirmed that silencing Rad1 expression aggravated the accumulation of DSBs and cell cycle arrest in AECII cells, whereas its overexpression alleviated DSB accumulation and cell cycle arrest.

**Conclusions:**

The accumulation of DSBs in AECII might be an important cause of alveolar growth arrest associated with BPD. Rad1 could be an effective target for intervention to improve this arrest in lung development associated with BPD.

## Introduction

Bronchopulmonary dysplasia (BPD) is the most common and serious complication in preterm babies, with rates reaching up to 45% in infants born at < 29 weeks gestational age (Eduardo et al. 2017; Sahni and Phelps 2011). The mortality and complication rates of preterm infants with BPD are significantly higher than those of preterm infants without BPD. It is often accompanied by long-term pulmonary and neurodevelopmental morbidities, which seriously affect the quality of life of the children (Stoll et al. 2015). The major pathological characteristics of BPD include arrested alveolar development, but the mechanism is still unclear. Many studies have confirmed that oxidative stress is closely related to BPD (Wang and Dong 2018). Within 1–2 days after birth, oxidative factors such as lipid hydrogen peroxide and lipid peroxidation products are present in plasma or bronchoalveolar lavage fluid of children with BPD (Fabiano et al. 2016). The increase in the levels of malondialdehyde, accompanied by a decrease in antioxidants, suggests an imbalance of the oxidation/antioxidation system in children with BPD in the early stages after birth (Simon-Szabo et al. 2021). Our group also confirmed such imbalances in the oxidation/antioxidant system in BPD and the lung injury induced by reactive oxygen species (ROS) at the animal and cell level (Hua 2004; Jian 2004). Oxidative stress products such as ROS can attack proteins, lipids, and DNA and cause oxidative damage. DNA damage is the most serious manifestation of oxidative stress. If not repaired in time, damaged DNA will seriously diminish the ability of both self-renewal and cell differentiation and even lead to gene mutations or cell apoptosis. Research shows that the accumulation of DNA damage is associated with a variety of diseases, including cancer, aging, stem cell dysfunction, metabolic syndrome and neurodegenerative diseases (Fr et al. 2016; Ishida et al. 2019; Pilzecker et al. 2019; Shah and Bennett 2017).

DNA double stranded breaks (DSBs) are one of the most serious and hazardous types of DNA damage because a single DSB is sufficient to kill a cell or destroy its genome integrity (Jackson and Bartek 2009). DSBs trigger the DNA damage response (DDR) leading to checkpoint-mediated cell cycle arrest, followed by DSB repair, and if the damage cannot be eliminated, the cell will enter permanent cell cycle arrest. Studies have shown that the imbalance between accumulation and repair of DSBs is an important pathogenic mechanism of neurodegenerative diseases and aging (Gorbunova and Seluanov 2016; Shanbhag et al. 2019; Thadathil et al. 2021; Ross and Truant 2017), but very little is known about the role of DSBs in BPD.

In the present study, we investigated DSBs using several approaches and provide compelling evidence of increased DSB accumulation and cell cycle arrest in BPD based on animal, primary cell, and in vitro data. A PCR array based on the DNA damage signaling pathway was used to study the expression of genes involved in DNA damage and repair in BPD, and the low expression of Rad1 was identified. We subsequently confirmed that silencing the expression of Rad1 could aggravate the accumulation of DSBs and cell cycle arrest in type II alveolar epithelial cells (AECIIs), whereas overexpression of Rad1 alleviated DSB accumulation and cell cycle arrest.

## Materials and methods

### Animal model

Healthy adult Sprague–Dawley (SD) rats were provided by the Animal Laboratory, Experimental Research Center of Shengjing Hospital affiliated to China Medical University. The Laboratory Animal Ethics Committee of Shengjing Hospital of China Medical University approved all animal procedures. Based on a modeling method that we previously established (Hou et al. 2015), newborn SD rats were randomly divided into a model group and a control group within 12 h after their natural birth. In the model group, the inhaled oxygen concentration was 85%, whereas the rats in the control group inhaled air (FiO_2_ 21%). Mother rats in the model group and the control group were exchanged daily to eliminate feeding differences and avoid oxygen poisoning.

### Primary AECII isolation, cell culture, transfection and grouping

As previously described (Hou et al. 2015), lung tissues were extracted, rinsed repeatedly and then digested with 0.25% trypsin EDTA (25200056, GIBCO, USA) for 30 min followed by with 0.1% collagenase I (C8140, Solarbio, USA). The rat alveolar type II epithelial cell line RLE-6TN (Mount Sinai School of Medicine, New York, NY) cells were randomly divided into a model group ((85% O_2_; 5% CO_2_) and a control group (21% O_2_; 5% CO_2_) and harvested after 12, 24, and 48 h of culture for the subsequent experiments. The siRNA sequence that targets rat Rad1 (siRad1) was designed and synthesized by Shanghai Hanbio Biotechnology Co. Ltd. (Shanghai, China) and transfected with Lipofectamine 2000 (Thermo Fisher Scientific, USA). The plasmid DNA clones for rat Rad1 and the empty vector were purchased from Shanghai GenePharma Co., Ltd. (Shanghai, China).

### Morphological observation of lung tissues

Rat lung tissues from the two groups were extracted on days 1, 3, 7, and 14 after birth. The lower lobe of the right lung was fixed in 4% paraformaldehyde for 48 h and then removed for dehydration, dewaxing, and paraffin embedding. The tissues were cut into 4-µm-thick paraffin sections and hematoxylin and eosin (H&E)-stained for morphological analysis. Radial alveolar counts (RACs) indicate the number of alveoli on the vertical line from the center of the respiratory bronchioles to the nearest fibrous septum (or pleura). H&E-stained sections were observed under 10 × magnification and the number of alveoli was counted 10 times (for each section); the average values were calculated to evaluate the degree of alveolar development.

### Immunofluorescence staining

Immunofluorescence staining was performed to detect phosphorylated H2A histone family member X (γ-H2AX) in the paraffin sections and RLE-6TN cells. The samples were treated with rabbit anti-γ-H2AX (1:300, ab81299, Abcam) primary antibodies overnight at 4 °C, washed and followed by incubation with the secondary antibody Donkey Anti-Rabbit IgG H&L Alexa Fluor^®^ 594 Conjugate (ab150076, Abcam) for 4 hours at room temperature. Double immunofluorescence staining was used to detect the expression of γ-H2AX in AECIIs and the combination of primary antibodies used was rabbit anti-γ-H2AX (1:300, ab81299, Abcam) and mouse anti-p180 (ribosome-binding protein 1) (1:100, ab24751, Abcam). The combination of secondary antibodies used was Donkey Anti-Rabbit IgG H&L Alexa Fluor^®^ 594 Conjugate (ab150076, Abcam) and Donkey Anti-Mouse IgG H&L Alexa Fluor^®^ 488 Conjugate (ab150105, Abcam).

### Western blotting

Protein lysates (30 µg per well) were separated on 4–12% bis–tris gels, blotted onto a polyvinylidene difluoride membrane, and probed with the indicated primary antibodies as follows: rabbit anti-γ-H2AX (1:5000, ab81299, Abcam), rabbit anti-p21 (1:1000, ab109199, Abcam); rabbit anti-Rad1 (1:500, 11726-2-AP, Proteintech), or mouse anti-β-actin (1:5000, 60008-1-Ig, Proteintech) and secondary antibodies were goat anti-rabbit IgG (H + L) (SA00001-2, Proteintech) or goat anti-mouse IgG (H + L) (SA00001-1, Proteintech). The membranes were visualized using an Alpha Ease RFC Imaging System (Alpha Innotech, San Leandro, CA, USA).

### Enzyme-linked immunosorbent assay (ELISA)

We used 8-OHdG ELISA Kits (EU2548, Fine Biotech, CHINA) to detect 8-hydroxy-2′-deoxyguanosine (8-OHdG) levels in the rat lung tissues. According to the instructions, 100 mg of rat lung tissue was added into 1 ml of cell lysate and crushed by ultrasound. The lysate was centrifuged at a low temperature and the resulting supernatant was used for testing. Fifty microliters of standard, blank, or sample was added to each well, and incubated at 37 °C for 45 min. Absorbance was measured at 450 nm using a Microplate Reader. 8-OHdG levels of each sample were calculated according to the standard curve.

### Comet assays

RLE-6TN cells were resuspended in PBS to adjust the concentration to 1 × 10^6^ cells/ml. A three-layer gel was prepared, after which, cell lysis, alkaline unwinding of the DNA, and single-cell electrophoresis was performed. The Comet Assay Software Project image analysis software was used to analyze the comet images, using commonly used indicators, specifically comet tail length, olive tail moment, and comet tail DNA content.

### PCR and PCR arrays

RNA was extracted using Trizol reagent (Takara, Japan), and mRNA was reverse transcribed using the TAKARA Reverse Transcription Kits. The primers were designed and synthesized by Sangon Biotech, China (Table 1). The results were analyzed using the relative gene expression (2^−ΔΔ^CT) method. Three rat pups from the model and control groups were randomly selected on the 14th day after modeling and lung tissues were extracted and dissolved in Trizol. We used the 96-well based rat DNA damage signaling pathway PCR array (PARN-029, SABiosciences, USA) to perform quantitative real-time reverse transcription PCR in the ABI PRISM 7900-HT machine (Applied Biosystems, USA). The rat DNA damage signal RT PCR array contains 96 wells, and the expression of 84 DNA damage signal pathway-related genes was detected (Table 2).

**Table 1:** Primer sequences used for quantitative PCR

Gene	Forward (5′–3′)	Reverse (5′–3′)
Rad1	AAGGAGGAGTAGTGACGGTCTGC	AGGAGACACGGTGATCTGTAGGAC
β-actin	CTGAAGTACCCCATTGAACACGGCA	ATCTGGGTCATCTTTTCACGGTTGG

**Table 2:** Gene table for 84 genes of DNA damage signaling pathway PCR array

ATM / ATR Signaling
ATM, Bard1, Brca1, Cdc25a, Chek1, Chek2 (Rad53), Csnk2a2, Fancd2, Hus1, Parp1 (Adprt1), Parp2,Rad1, Rad17, Rad50, Rad9, Rnf8, Smc1a (Smc1l1), Topbp1, Tp53 (p53) DNA Damage & Repair Nucleotide Excision Repair (NER): Brca2, Ddb2, Dclre1a, Ercc1, Ercc2 (Xpd), Fancc, Lig1, Nthl1, Ogg1,Pcna, Pold3, Pole, Rpa1, Sirt1, Tp53 (p53), Xpc Base Excision Repair (BER): Apex1, Fen1, Lig1, Mbd4, Mpg, Nthl1, Ogg1, Parp1 (Adprt1), Parp2, Pcna,Pole, Tp53 (p53), Ung, Xrcc1, Wrn Mismatch Repair (MMR): Abl1, Exo1, Mlh1, Mlh3, Msh2, Msh3, Pcna, Pms1, Pms2 Double-Strand Break (DSB) Repair: Atm, Blm, Brca1, Brca2, Chek1, Hus1, Lig1, Mlh1, Mre11a, Nbn (Nbs1), Prkdc, Rad50, Rad51, Rad52, Rpa1, Tp53bp1, Xrcc2, Xrcc6 (G22p1) Other DNA Repair Genes: Atrx, Bard1, Chek2 (Rad53), Fanca, Fancd2, Fancg, Gadd45a, Gadd45g, Mgmt (Agt),Polh, Poli, Pttg1, Rad1, Rad17, Rad18, Rad21, Rad51c, Rad51b, Rad9, Rev1, Rnf8, Smc1a, Smc3, Sumo1, Topbp1, Wrnip1 Apoptosis Abl1, Atm, Bard1, Bax, Bbc3, Brca1, Cdkn1a (Cip1,Waf1), Chek2 (Rad53), Csnk2a2, Ppp1r15a (Gadd34),Prkdc, Rad21, Rad9, Sirt1, Terf1, Tp53 (p53) Cell Cycle Atm, Cdc25a, Cdc25c, Cdkn1a (Cip1,Waf1), Chek1, Chek2(Rad53), Ddit3 (Gadd153,Chop), Mif, Ppm1d (Wip1), Ppp1r15a (Gadd34), Terf1, Tp53 (p53)

### Cell cycle analysis

Cells were fixed in pre-cooled 75% ethanol and Phosphate Buffered Saline (PBS). Then, the cells were rinsed with PBS, and propidium iodide was added to analyze the cell cycle distribution via flow cytometry according to the manufacturer’s protocols. The results were analyzed using CELL Quest software (Becton Dickinson, USA).

### Statistical analysis

Data analysis was performed using GraphPad Prism version 8.0 (GraphPad software). All data are expressed as the mean ± standard deviation (χ ± s). Comparisons between the model group and the control group were analyzed using independent-sample t-tests or Mann–Whitney test. Differences among multiple groups were analyzed using one-way ANOVA. Differences with *p* values < 0.05 were considered statistically significant.

## Results

### Accumulation of DSB damage and increased expression of p21 in lung tissue of BPD rats

**Fig. 1:**
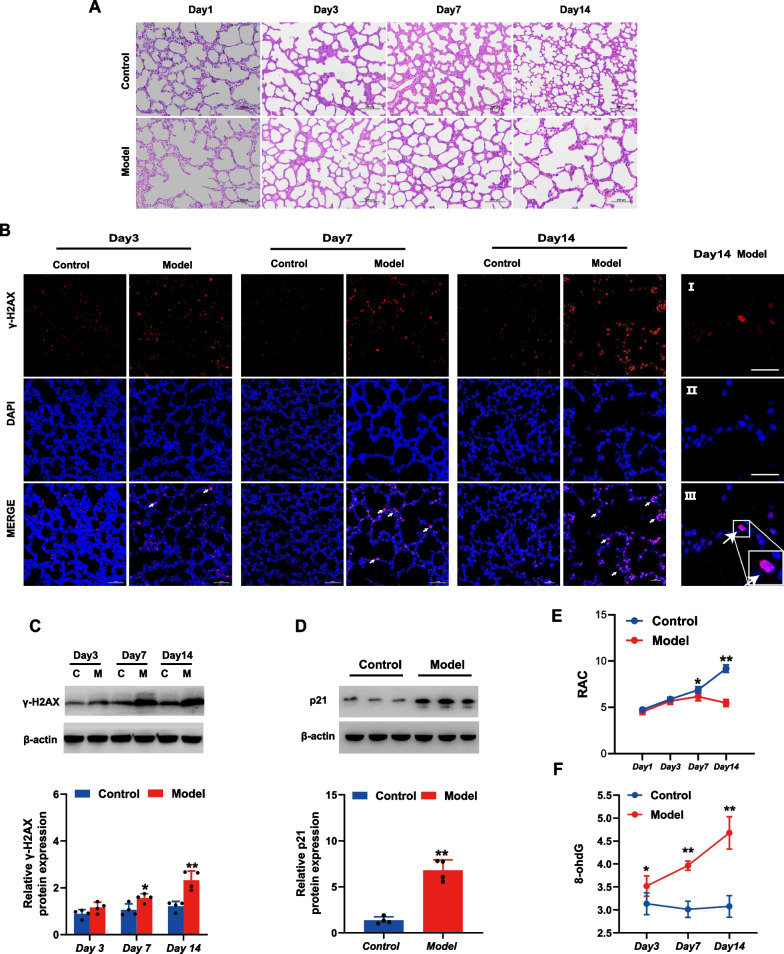
Accumulation of DNA double-strand breaks (DSBs) and increased expression of p21 in lung tissue of bronchopulmonary dysplasia (BPD) rats. **A** H&E staining visualized with a light microscope to observe morphological changes in lung tissue (scale bar = 100 μm). **B** Immunofluorescence staining of γ-H2AX (red) and DAPI (blue) in rat lung tissue in model and control groups at different time points (3, 7 and 14 days). (Scale bar = 50 μm.) (I–III) Enlarged image of γ-H2AX. Scale bar = 10 μm. **C** Expression levels of γ-H2AX in rat lung tissue in model and control groups at different time points (3, 7 and 14 days) detected by western blot. **D** Expression levels of p21 in rat lung tissue in model and control groups at 14 days detected by western blot (**E**) Comparison of Radial alveolar counts (RAC) for lung tissue between the two groups. **F** Level of 8-OHdG in rat lung tissue in model and control groups (C = control group, M = model group). *p < 0.05, **p < 0.01

H&E staining revealed that alveolarization in the control group gradually improved with increasing age (Fig. 1A) whereby the number of alveoli was increased and all were of the same size and very regular in shape as well as secondary alveolar septa. The rats in the model group (exposed to 85% O_2_) showed a significant delay in alveolar development. On the 7th day, the number of alveoli decreased and the size increased. On the 14th day, a further reduction in the number of alveoli, with more pronounced uneven sizes and reduced as well as blunted secondary alveolar septa was observed, demonstrating the simplification of the alveolar structure. The RAC value is an important indicator for evaluating lung development (Herring et al. 2014). A larger RAC value indicates more mature lung development. As shown in Fig. 1E, the RAC value of the model group decreased significantly from the 7th day (p < 0.05), and the difference was more significant on the 14th day (p < 0.01), suggesting the arrest of alveolarization after hyperoxia. 8-OHdG represents a sensitive marker of oxidative DNA damage. The ELISA results (Fig. 1F) showed that compared with levels in the control group, the level of 8-OHdG in the model group significantly increased from the third day, and the difference was more pronounced at days 7 and 14 (p < 0.01). This suggested an increase in DNA oxidative damage at the early stages, which accumulated with time. The phosphorylation of histone variant H2AX at residue Ser139 (γ-H2AX) is an early and important cellular response to the induction of DSBs, and the detection of this phosphorylation event has emerged as a reliable and sensitive molecular marker for monitoring DSBs (Siddiqui et al. 2015). Immunofluorescence and western blotting analyses (Fig. 1B, C) showed that in the model group, γ-H2AX levels increased starting on day 3 and were significantly higher on day 7 (p < 0.05) and more pronounced on day 14 (p < 0.01), suggesting the accumulation of DSBs in the lungs of BPD rats over time. In addition, we examined the expression of p21, a cell cycle arrest-related protein. As shown in Fig. 1D, compared to the control group, the p21protein levels in the model group were significantly increased (p < 0.01).

### Accumulation of DSBs and cell cycle arrest in the AECIIs of rats with BPD

**Fig. 2:**
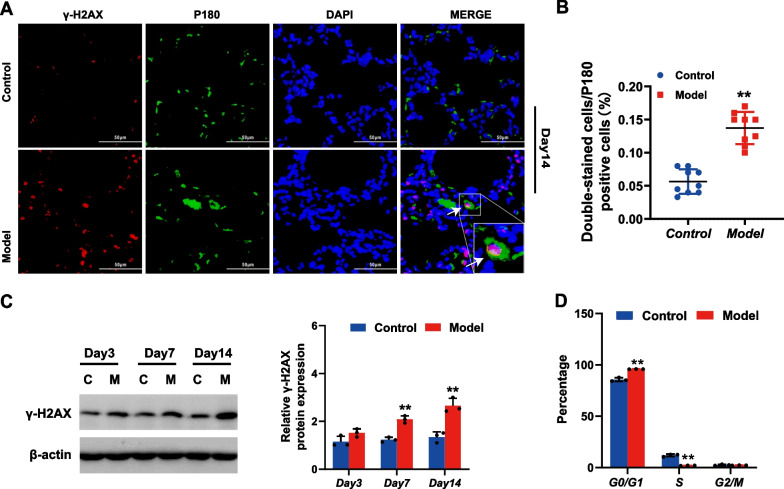
Accumulation of DNA double-strand breaks (DSBs) and cell cycle arrest in the AECIIs of rats with bronchopulmonary dysplasia (BPD). **A** Immunofluorescence double staining of γ-H2AX and p180 in rat lung tissue in model and control groups at 14d. (Scale bar = 50 μm, Red fluorescence-labeled γ-H2AX, green fluorescence-labeled p180 represents AECII, blue fluorescence-labeled DAPI represent the nucleus). **B** The percentage of double-stained cells/p180 positive cells in model and control groups. **C** Expression level of γ-H2AX in primary AECIIs in model and control groups at different time points (3, 7 and 14 days) detected by western blot. **D** Cell cycle analyses of primary AECII in model and control groups at 14 days (C = control, M = model). *p < 0.05, **p < 0.01

To further reveal the level of DSBs in AECIIs, we first detected the co-expression of γ-H2AX and p180, which are biomarkers of AECIIs, in the lung tissue of rats at 14 days using double immunofluorescence staining. As shown in Fig. 2A, co-expression of p180 and γ-H2AX could be seen in the lung tissue in the model group, whereas in the control group, there was little co-expression. The double-stained cells were counted (Fig. 2B) and the percentage of double-stained cells /p180 positive cells in the model group was significantly higher than that in the control group (p < 0.01). To further explore the DSBs in the AECIIs of rats with BPD, we extracted primary AECIIs at different rat ages (3, 7, and 14 days) and performed western blotting experiments to quantitatively detect the level of γ-H2AX in primary AECIIs at different time points. The results (Fig. 2C) showed that compared with the control group, the level of γ-H2AX in the primary AECIIs of the model group increased in model rats aged 3 days old and was significantly unregulated at 7 day old rats (p < 0.01). The difference was more pronounced at 14 days (p < 0.01), suggesting an accumulation of DSBs in the AECIIs of rats with BPD that progressively increased with time. The DNA damage checkpoints were initiation to respond to the accumulation of DSB and enter cells into a viable but non-replicative state. Therefore, to assess the cell cycle arrest, we examined we assessed the cell cycle distribution. To further verify cell cycle arrest in BPD, flow cytometric analysis was used to detect the cell cycle distribution of the primary AECIIs in both control and BPD neonatal rats. When compared to the control group, the percentage of cells in the G0/G1 phase in the model group was significantly increased (p < 0.01; Fig. 2D), whereas the percentage of cells in the S phase was significantly reduced (p < 0.01; Fig. 2D), suggesting increased cell cycle arrest in the AECIIs of rats with BPD.

### Accumulation of DSBs in cultured AECIIs after exposure to hyperoxia

**Fig. 3:**
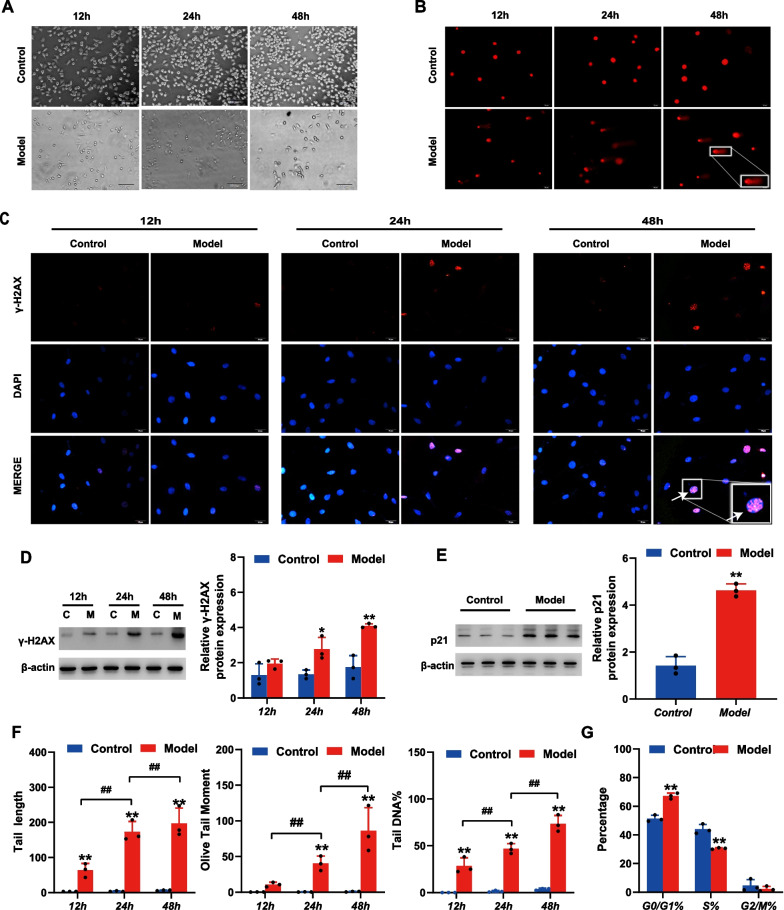
Accumulation of DNA double-strand breaks (DSBs) in cultured AECII cells after exposure to hyperoxia. **A** Cell status detected under the microscope. **B** DNA damage in cultured AECII cells after exposure to hyperoxia and normoxia at different time points (12, 24 and 48 h) detected by comet assay (Scale bar = 20 μm). **C** Immunofluorescence staining of γ-H2AX in cultured AECII after exposure to hyperoxia and normoxia at different time points (12, 24 and 48 h). (Scale bar = 10 μm, Red fluorescence-labeled γ-H2AX, blue fluorescence-labeled DAPI represent the nucleus). **D** Expression levels of γ-H2AX in cultured AECII after exposure to hyperoxia and normoxia at different time points (12, 24 and 48 h) detected by western blot. **E** Expression levels of p21 in cultured AECII after exposure to hyperoxia and normoxia at 48 h as detected by western blot. **F** Comet tail length, olive tail moment, and percentage of tail DNA content in cultured AECII cells. **G** Cell cycle analyses of cultured AECII in model and control groups at 48 h. (C = control, M = model). **p < 0.01; ^##^p < 0.01

To further explore the DSBs in AECIIs associated with BPD, we established an in vitro model using the RLE-6TN cell line exposed to hyperoxia or normoxia, to observe DSBs at different time points (12, 24, and 48 h) under oxidative stress. Cell status was observed under a microscope. The results (Fig. 3A) showed that cells in the control group grew well, and the number of cells continued to increase over time. Compared with the control group, the number of cells in the model group began to decrease at 12 h, and deteriorated at 24 and 48 h. Immunofluorescence and western blotting analyses (Fig. 3C, D) showed that the level of γ-H2AX in the model group significantly increased from 24 h (p < 0.05) compared to the control group, and the difference was more pronounced at 48 h (p < 0.01). A comet assay detected minimal migration of DNA following exposure to normoxia. In contrast, significant migration of DNA occurred from the nucleus at 12 h, forming a “comet tail.” Relative to the control group, comet tail length, olive tail moment, and percentage of tail DNA in the model group increased from 12 h (p < 0.01), and the difference was more pronounced at 24 and 48 h (p < 0.01; Fig. 3B, F). Next, we detected the expression of p21 and the cell cycle distribution at 48 h in the two groups of cells. The western blots showed that compared with the control group, p21 protein levels in the model group were significantly increased (p < 0.01; Fig. 3E). Moreover, as shown in Fig. 3G, the percentage of cells in the G0/G1 phase in the model group was significantly increased (p < 0.01) compared with that in the control group, whereas the percentage in S phase was significantly reduced (p < 0.01), suggesting aggravation of cell cycle arrest in the AECIIs after exposure to hyperoxia.

### Gene expression analysis by DNA damage signaling pathway-based PCR array

**Fig. 4:**
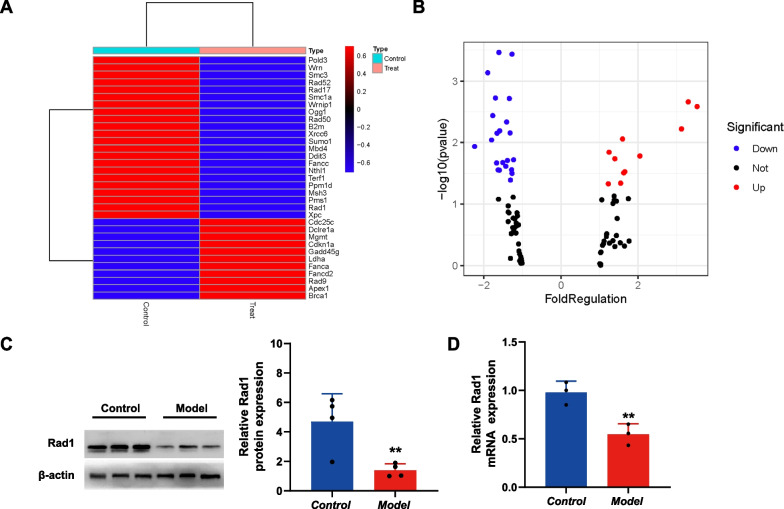
DNA damage signaling pathway-based PCR array analysis of DNA damage. **A** Heat map of the relative expression levels. **B** Volcano plot of the relative expression levels. Genes were considered to be differentially expressed if there was a > 1.5-fold difference in gene expression with a threshold p value of < 0.05. Red represents up-regulated genes while blue represents down-regulated genes. **C** The expression levels of Rad1 protein in lung tissue of the model group were significantly down regulated. **D** Expression levels of Rad1 mRNA in lung tissues of the model group were significantly down regulated **p < 0.01

To study the expression of genes involved in DNA damage and repair in BPD, a PCR array based on the DNA damage signaling pathway, comprising 84 relevant genes, was used with lung tissues of rats with BPD and controls. Genes were considered differentially expressed if there was a > 1.5-fold difference in gene expression, with a threshold p value < 0.05. Of the 84 genes in the DNA damage signaling pathway PCR array, 19 genes had altered expression. Eight genes, Brca1, Cdc25c, Cdkn1a, Dclre1a, Fanca, Fancd2, Gadd45g, and Mgmt, were overexpressed, whereas 11 genes exhibited reduced expression, namely Ddit3, Fancc, Mbd4, Msh3, Nthl1, Pms1, Ppm1d, Rad1, Rad52, Smc3, and Wrn (Table 3 and Fig. 4A, B). Rad1 is an important protein for DSB repair and the PCR array results showed that its expression was decreased in the model group. Then, real-time PCR and western blot assays were used to verify the microarray results. Both analyses showed that compared with those in the control group, the expression levels of Rad1 protein and mRNA in the lung tissues of the model group were significantly downregulated (Fig. 4C, D). This indicated that the inhibition of Rad1 expression might be an important factor in the accumulation of DSBs.

**Table 3:** Gene expression analysis by DNA damage signaling pathway-based PCR array

Gene bank	Symbol	Gene function	Fold regulation model/control		p value
			Up	Down	
NM_012514	Brca1	ATM / ATR signaling	1.55		0.045666
NM_001107396	Cdc25c	Cell cycle	3.13		0.005991
NM_080782	Cdkn1a	Cell cycle	3.53		0.002586
NM_001106201	Dclre1a	Nucleotide excision repair	1.60		0.008727
NM_001108455	Fanca	Fanconi anemia pathway	1.63		0.031054
NM_001001719	Fancd2	Fanconi Anemia pathway	1.66		0.029674
NM_001077640	Gadd45g	Apoptosis	2.05		0.016492
NM_012861	Mgmt	DNA repair	3.30		0.002173
NM_012557	Fancc	Fanconi anemia pathway		− 1.67	0.021384
NM_024134	Ddit3	Apoptosis		− 1.70	0.001880
XM_001059437	Mbd4	Base excision repair		− 1.80	0.009073
XM_001065837	Msh3	Mismatch repair		− 2.24	0.011564
NM_001105728	Nthl1	Base excision repair		− 1.63	0.027792
NM_001009535	Pms1	Mismatch repair		− 1.61	0.000344
NM_001105825	Ppm1d	Negative feedback regulation of DNA damage repair		− 1.51	0.021103
NM_001106419	Rad1	Double-strand break repair		− 1.90	0.000737
NM_001106617	Rad52	Double-strand break repair		− 1.77	0.003632
NM_031583	Smc3	Double-strand break repair		− 1.59	0.006429
XM_214361	Wrn	Base excision repair		− 1.60	0.028017

*p* value of < 0.05, fold difference > 1.5

Relative expression profiles of genes associated with the DNA damage signaling pathway in model and control rat lung tissues. Genes were considered to be differentially expressed if there was a > 1.5-fold difference in gene expression, with a threshold* p* value of < 0.05

### Rad1 attenuates DSBs and cell cycle arrest in AECIIs

**Fig. 5:**
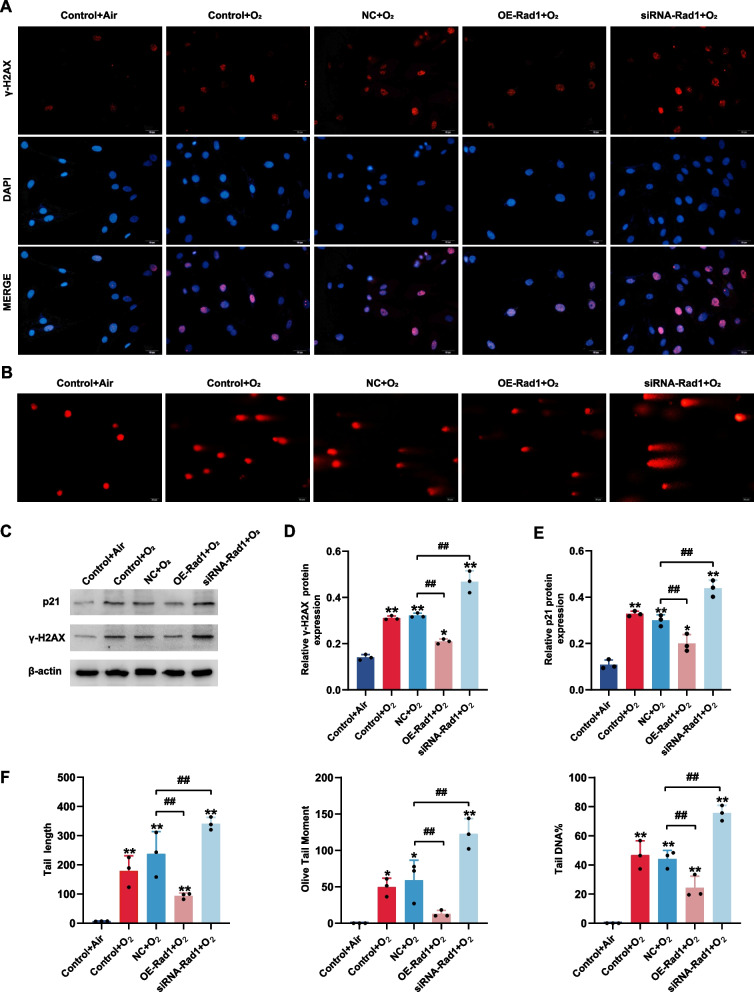
Rad1 attenuates DNA double-strand breaks (DSBs) and cell cycle arrest in AECII. RLE-6TN cells were randomly divided into five groups: a non‐interference air group (Control + 48hAir), a non‐interference hyperoxia group (Control + 48hO2), a blank-transfection hyperoxia group (NC + 48hO2), a siRNA-Rad1 transfection hyperoxia group (siRNA-Rad1 + 48hO2), and a Rad1 overexpression hyperoxia group (OE-Rad1 + 48hO2). **A** Immunofluorescence detection of γ-H2AX expression in RLE-6TN cells transfected with Rad1 overexpression or knockdown constructs. (Scale bar = 10 μm, Red fluorescence-labeled γ-H2AX, blue fluorescence-labeled DAPI represent nucleus). **B** Comet assay of DNA damage in RLE-6TN cells transfected with Rad1 overexpression or knockdown constructs (Scale bar = 20 μm). **C**–**E** Western blot detection of γ-H2AX and p21 expression in RLE-6TN cells transfected with Rad1 overexpression or knockdown constructs (C = control, M = model). **F** The comet tail length, the olive tail moment, and the percentage of tail DNA content in RLE-6TN cells transfected with Rad1 overexpression or knockdown constructs

**Fig. 6:**
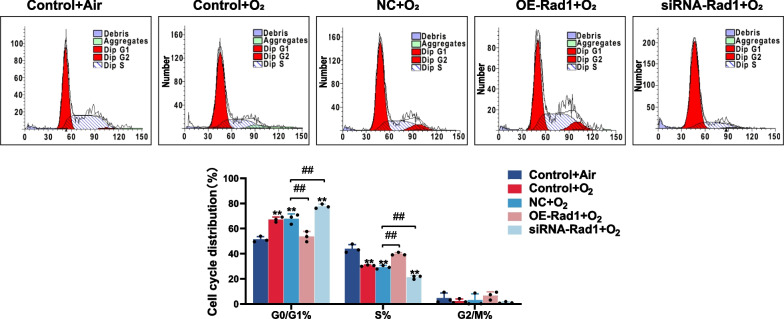
Cell cycle analyses of RLE-6TN cells transfected with Rad1 overexpression or knockdown constructs. *p < 0.05, **p < 0.01, ^#^p < 0.05, ^##^p < 0.01

To confirm our hypothesis, RLE-6TN cells were transfected with Rad1 overexpression or knockdown constructs before the cells were exposed to hyperoxia or normoxia, and DSB indicator levels were measured at 48 h. RT-qPCR results showed that Rad1 was successfully overexpressed and inhibited. The western blot results (Fig. 5C, D) showed that the expression of γ-H2AX was significantly reduced after the overexpression of Rad1 compared to that in the empty vector group (p < 0.01); however, the expression of γ-H2AX was significantly increased in the presence of Rad1 knockdown (p < 0.01), which was consistent with the immunofluorescence results (Fig. 5A). The results of the comet assay showed that the comet tail length, olive tail moment, and percentage of tail DNA were significantly reduced after the overexpression of Rad1 compared to the empty vector group but were significantly increased after Rad1 knockdown (Fig. 5B, F; p < 0.01). These results indicated that DSBs in ACEIIs were alleviated after the overexpression of Rad1 but aggravated after Rad1 knockdown. We further analyzed the effect of Rad1 overexpression/knockout on cell cycle arrest in RLE-6TN cells. The western blot results (Fig. 5C, E) showed that the expression of p21 significantly decreased in the background of overexpressed Rad1 compared to that in the empty vector group (p < 0.01) but increased after Rad1 knockdown. The results of flow cytometry analysis (Fig. 6) showed that after the overexpression of Rad1, the percentage of cells in S phase was significantly increased and the percentage of cells in G0/G1 phase was significantly decreased compared to levels in the empty vector group (p < 0.01), indicating the removal of cell cycle arrest. However, cell cycle arrest was aggravated after Rad1 knockdown (p < 0.01).

## Discussion

BPD is the most common and serious complication of preterm infants. Its main pathological feature is alveolar development arrest, but the mechanism is still unclear. Based on previous reports of the presence of oxidative stress and DNA oxidation metabolites, this study used a neonatal rat model with hyperoxia-induced BPD to confirm the continuous accumulation of DSBs and cell cycle arrest in rat ACEIIs with BPD. Then, we used a PCR array based on the DNA damage signalling pathway to study the expression of genes involved in DNA damage and repair in BPD and Rad1 was screened based on its low expression. Moreover, we confirmed at the cell level that silencing the expression of Rad1 can aggravate the accumulation of DSBs and cell cycle arrest in AEC II, whereas the overexpression of Rad1 could reduce the accumulation of DSBs and cell cycle arrest in AEC II.

The results of this study show that the oxidative DNA damage marker 8-ohdG in the lung tissue of BPD rats is significantly increased, which is consistent with the clinical results (Hsiao et al. 2017, 2021; Joung et al. 2011; Tokuriki et al. 2015). The 8-ohdG content in the lung tissue of BPD rats continued to increase with age, proposing the accumulation of DNA damage in the lung tissue of BPD rats over time. DSBs are the most serious type of DNA damage, but current research on DSBs in BPD is still lacking. γ-H2AX is a classic marker for the early recognition of DSB-associated damage, and the persistence of γ-H2AX indicates the continuous accumulation of unrepaired DSBs because γ-H2AX is immediately dephosphorylated by Protein Phosphatase 2 (PP2A) after DSB repair (Siddiqui et al. 2015). A previous study showed that Aurora-A inhibits p21 (waf1/cip1) and p27 (cip/kip) but enhances Plk1, CDC25, CDK1, and cyclin B1 to repeal cell cycle checkpoints and promote cell cycle progression. Aurora-A also suppresses BRCA1, BRCA2, RAD51, poly (ADP ribose) polymerase (PARP), and γ-H2AX to deregulate the DNA damage response (Wang et al. 2014). In this study, fluorescence and western blot detection of γ-H2AX confirmed that DSBs occurred in the lung tissue of BPD rats, and DSBs continued to accumulate with age. Subsequently, we confirmed that levels of p21 continued to increase in the lung tissue of BPD rats with age, suggesting that BPD is associated with an accumulation of DSBs and an associated cell cycle block in lung tissue. Cyclin-dependent kinase (CDK) inhibitor p21, also known as p21waf1/cip1 or P21/CDKN1A, is a well-known inhibitor of the cell cycle and can arrest cell cycle progression at G1/S and G2/M transitions by inhibiting CDK4, 6/cyclin-D, and CDK2/cyclin-E. Studies have shown that continuous accumulation of DSB damage can induce DNA Damage Response (DDR) to activate p21 and induce permanent cell cycle arrest (Dutto et al. 2016; He et al. 2020; Karimian et al. 2016). A study by Das et al. also confirmed the upregulation of p21 expression in the lung tissue of baboons with BPD (Das et al. 2004). Lande et al. also confirmed that the alveolar hypoplasia caused by hyperoxia exposure in neonatal mice is related to cell cycle arrest and decreased Histone Deacetylase activity caused by the upregulation of p21 (Londhe et al. 2011). These results are consistent with the finding of this study, and collectively provide evidence to support our hypothesis that the cell cycle arrest induced by the accumulation of DSBs might be an important cause of BPD-associated alveolar growth arrest.

AECIIs are progenitor cells in the alveolar epithelium. They can self-proliferate and also differentiate into type I alveolar epithelial cells, which is important in the maintenance of alveolar structure and damage repair during lung injury. Therefore, we extracted primary type 2 alveolar epithelial cells from rats of different ages in the BPD and control groups and noted an increase in the extent of DSBs in AECII cells with BPD, which accumulated over time. We also conducted an in vitro cell hyperoxia experiment and the results demonstrated that the levels of DSBs in the RLE-6TN cell line increased after 12 h of exposure to hyperoxia, with breaks accumulating over increasing exposure time, which was consistent with the results of the in vivo experiments. The cell cycle is a series of tightly regulated molecular events controlling DNA replication and mitosis. Every cell cycle, individual metazoan cells have the opportunity to adopt one of two mutually-exclusive proliferation-related fates: continued cell cycle progression or cell cycle exit. Cell cycle exit encompasses multiple distinct states including permanent arrests associated with terminal differentiation or senescence and reversible exit known as quiescence (G0). Flow cytometric analysis was used to detect the cell cycle distribution of the primary AECIIs and RLE-6TN. The percentage of cells in the G0/G1 phase in the model group was significantly increased, which suggesting increased cell cycle arrest in the AECIIs of rats with BPD. The accumulation of DSBs and cell cycle arrest in AECII with BPD were also confirmed.

Unrepaired DSB accumulation activates the p53-p21 pathway and induces permanent cell cycle arrest, suggesting that the imbalance between DSB accumulation and repair might be the key reason for the obstruction of alveolar development with BPD. This has also been linked to the processes related to neurodegenerative disorders and aging (Gorbunova and Seluanov 2016; Shanbhag et al. 2019; Thadathil et al. 2021; Ross and Truant 2017). p53 facilitates DNA repair by halting the cell cycle to allow time for the repair machinery to restore genome stability. In addition, p53 also directly impacts the activity of various DNA-repair systems. Moreover, cells have evolved a mechanism called DDR to sense DSBs and to transmit and amplify a series of signals that promote the repair of DNA damage (Ishida et al. 2019; Pilzecker et al. 2019; Shah and Bennett 2017). Cells defective in these mechanisms generally display heightened sensitivity toward DSB agents and the accumulation of such defects causes human diseases. The signal pathway RT^2^Profiler PCR Array relies on RT-qPCR and the ability of microarrays to quantify the simultaneous expression of multiple genes. Therefore, a PCR array based on the DNA damage signaling pathway which includes 84 related genes, was used to assess changes in gene expression in the lung tissue of BPD and control group rats. There were 19 differentially expressed genes, of which, eight were highly expressed in the model group, among these, BRCA1, p21WAF1/CIP1, Cdc25c, and Gadd45 are related to cell cycle arrest. Cdkn1a (p21) showed the most pronounced changes in gene expression, with 3.53-fold change relative to the control group. This further confirmed the activation of cycle-related proteins after DSB accumulation in BPD and supported the idea that cell cycle arrest is an important factor in alveolar dysplasia.

We noted the low level expression of Rad1, a protein associated with DSB repair. Western blot and PCR analysis verified that Rad1 protein and mRNA expression levels in lung tissue of the model group were significantly downregulated. It is suggested that insufficient DSB repair in lung epithelial cells of BPD rats caused by the inhibition of Rad1 expression might be an important cause of alveolar dysplasia. Rad1 is one of the constituent proteins of the 9-1-1 checkpoint complex (Rad9, Rad1, and Hus1 proteins) and plays a key role in DNA repair and the cell cycle checkpoint (Rowley et al. 1992; Shimada et al. 2002). Studies have found that compared with other organs, mRNA levels of these three genes are significantly higher in lung tissue, which might be related to the increased demand for DNA damage repair resulting from exposure to high levels of oxidative stress (Zhang et al. 2015). In addition, Rad1 can also act on DNA damage independently of the 9-1-1 complex. Rad1 is a key element in the fission yeast *Schizosaccharomyces pombe *that mediates multiple cellular responses to DNA damage and in particular regulates cell cycle checkpoints. The results of siRNA-driven human Rad1 knockdown showed that Rad1 is an important element for cell growth and is necessary for the recovery of DNA synthesis following hydroxyurea treatment (Bao et al. 2004). Zhang et al. confirmed the defective S/M and G2/M cell cycle checkpoint controls and deficient cellular DNA damage responses caused by Rad1 targeted deletion in mice (Zhang et al. 2011). However, research on Rad1 in BPD is still lacking. In this study, we confirmed that after silencing the expression of Rad1, the accumulation of DSBs in cells increases after exposure to hyperoxia and following an increase in the expression of p21 and cell cycle arrest. However, overexpression of Rad1 alleviated the accumulation of DSBs and cell cycle arrest, which suggested that Rad1 is an effective target for intervention to improve the arrest in lung development associated with BPD.

## Conclusions

In summary, this study confirmed the accumulation of DSBs and cell cycle arrest in AECIIs of BPD rats, which increased with age, suggesting that the accumulation of DSBs in these cells might be an important cause of the alveolar growth arrest associated with BPD. Rad1 was downregulated in the BPD group and it was confirmed that the upregulation of Rad1 could reduce DSB damage and cell cycle arrest in AECIIs. This also suggested that the downregulation of Rad1 expression inhibits DSB damage repair in these cells, which leads to the accumulation of damage, and might be a key factor in the alveolar growth arrest associated with BPD. This also suggested that Rad1 could be an effective target for intervention to reduce the obstruction in lung development associated with BPD.

## Data Availability

Not applicable.

## Abbreviations

BPD: Bronchopulmonary dysplasia
ROS: Reactive oxygen species
DSBs: DNA double stranded breaks
DDR: DNA damage response
AECIIs: Type II alveolar epithelial cells
CDK: Cyclin-dependent kinase
